# Enhancing medication literacy through a telepharmacy call center in Israel: consultation overview and patient satisfaction

**DOI:** 10.1186/s13584-025-00686-4

**Published:** 2025-05-01

**Authors:** Ran Nissan, Rana Cohen, Maria Hurgin, Hen Popilski, Khaleel Zahalka, Merav Ben Natan, Eyal Schwartzberg

**Affiliations:** 1 Pharmaceutical Society of Israel, Tel Aviv, Israel; 2 Division of Clinical Pharmacy, Institute for Drug Research, School of Pharmacy, The Hebrew University, Jerusalem, Israel; 3 Pat Matthews Academic School of Nursing, Hillel Yaffe Medical Center, Hadera, 26736 Israel; 4 School of Pharmacy, Community Clinical Pharmacy and Regulatory Management MSc Program, Ben Gurion University, Beer Sheva, Israel

**Keywords:** Telepharmacy, Telehealth, Pharmaceutical counseling, Healthcare access, Patient satisfaction

## Abstract

**Background:**

Telepharmacy, the use of telecommunications technology to facilitate pharmacy services, has emerged as an integral component of telehealth, particularly during the COVID-19 pandemic. In Israel, the shortage of pharmacists nationwide has led to longer wait times and reduced consultation opportunities at community pharmacies. In response, the Pharmaceutical Society of Israel (PSI) established a telepharmacy call center to provide free pharmaceutical consultations to the public. This study aimed to describe the framework of this center, the types of pharmaceutical consultations and patient satisfaction with the service.

**Methods:**

This cross-sectional observational study analyzed unidentified data from 1,542 ambulatory patient inquiries to the PSI telepharmacy call center between October 2022 and June 2023. The consultations were categorized into clinical, logistical, and patient rights-related inquiries. A satisfaction survey was conducted among a representative sample of callers.

**Results:**

The majority of inquiries (93.3%) were received via telephone, with the 65–85 age group accounting for 38.4% of callers. A small proportion of inquiries were submitted via email, either exclusively or in combination with a telephone communication. Clinical inquiries comprised 89% of the total, with the most common topics being drug interactions (26.7%), general drug usage guidance (17.8%), and inquiries about drug side effects (16.4%). The patient satisfaction survey revealed that 87% of respondents strongly agreed that the pharmacists demonstrated empathy and attentiveness, and 93.5% were satisfied with the responses provided. The overall service rating was 8.9 out of 10, and 94.1% of respondents were willing to recommend the call center to others.

**Conclusion:**

This study highlights the value and feasibility of operating a national telepharmacy call center in Israel, addressing the diverse pharmaceutical needs of the public, particularly the elderly population. The high satisfaction levels among callers underscore the potential for such initiatives to enhance access to comprehensive pharmaceutical consultation and improve medication management.

**Supplementary Information:**

The online version contains supplementary material available at 10.1186/s13584-025-00686-4.

## Background

Societal shifts and technological advancements promoted alternative approaches to delivering pharmacy services. The term “telepharmacy” refers to the use of telecommunications technology to facilitate or enable the delivery of high-quality pharmacy services in situations where the patient or healthcare team does not have direct (in-person) contact with pharmacy staff [1, 2].

The practice of pharmacy within the realm of telehealth, as defined by the American Society of Health System Pharmacists (ASHP), may encompass a wide array of activities, including but not limited to: comprehensive drug therapy management, management of chronic disease states, patient assessment, identification and monitoring of adverse drug effects, patient clinical counseling, outcome evaluation, analysis of health system data, collaboration with other healthcare providers within the system and provision of drug information [3].

Telepharmacy is one approach to improving the quality of patient care. Governments, healthcare leaders, and the public at large have expectations for convenient and timely access to care, patient safety and improved health outcomes, financial sustainability, and appropriate scopes of practice for healthcare professionals. Telepharmacy can address all of these expectations by strengthening the profession’s alignment with healthcare needs and responding to stresses on the healthcare system [1].

Notably, telepharmacy, as an integral component of telehealth, has become increasingly evident amid the ongoing COVID-19 public health emergency. Multiple clinics, hospitals, and healthcare providers have embraced telepharmacy practices, and even after pandemic subsided, they have continued to implement it. This persistence can be attributed to the paramount goal of safeguarding the well-being of both patients and healthcare professionals [4–8].

The shortage of Israeli pharmacists nationwide has worsened in recent years [9], leading to longer wait times and reduced consultation opportunities at community pharmacies. As a result, patients are discouraged from seeking information about their treatment due to inadequate privacy and overcrowded waiting areas, prompting them to rely on nonmedical online sources and social media for information. This pattern particularly impacts the elderly, who often have restricted access to such digital resources.

In response, the Israeli Ministry of Health has previously expressed its commitment to advancing remote health services to enhance patient care. This includes the objective of improving pharmaceutical services through the implementation of telepharmacy [10].

To address this crucial gap and deliver comprehensive pharmaceutical consultation, the Pharmaceutical Society of Israel (PSI), a nonprofit organization, has partnered with The Israeli Society for Patients’ Rights [11] to launch a pharmacist-operated call center for the general public. This initiative aims to offer detailed, private, and thorough pharmaceutical consultation services.

The current study aim is to describe the framework of this telepharmacy call center operated by pharmacists from the Pharmaceutical Society of Israel (PSI) who provide telepharmacy services free of charge for the public via telephone and e-mail consultations and to describe the type of pharmaceutical consultation that was carried out as part of the pharmaceutical consultation as well as the satisfaction of the patients who contacted the call center.

## Methods

### Database

In this retrospective cross-sectional observational study we used unidentified data of ambulatory patients calling the PSI telepharmacy call center, from October 2022 to June 2023. Each call was collected and documented using a collaborative Microsoft 365 Office Excel file. The database recorded the nature, quality, and patient perception of the call center service, along with categorizing the consultations and their outcomes.

The Hillel Yaffe Medical Center Institutional Review Board (#HYMC-0122-23) approved the study (October 26, 2023) which was conducted in accordance with the principles set forth in the Helsinki Declaration. Informed consent was not required as this was a retrospective study, and patient data were obtained from the PSI database.

### Pharmacist training

Pharmacists underwent specialized training to develop the necessary skills to address patients’ inquiries regarding various aspects of pharmaceutical counseling. This training emphasized navigating and extracting relevant information from authoritative medical databases, including the Israeli Ministry of Health drug registry, Micromedex, UpToDate, and Reprotox. These resources were essential for providing accurate and up-to-date information on medication regimens, indications, contraindications, drug-drug interactions, and other relevant details.

Additionally, pharmacists were trained in algorithms for conducting inventory checks and were educated on the intricacies of patients’ medical rights and access to medications.

The comprehensive four-hour training session included instruction on effective communication strategies for extracting crucial medical information during conversations when medical records are unavailable. Special emphasis was placed on cultivating empathy and allowing patients ample time to express their concerns, thereby fostering a supportive and understanding environment to effectively address their needs.

### Call center operation and Documentation

The telepharmacy call center was staffed with ten pharmacists who worked three-hour shifts, five days a week, with two pharmacists per shift. The shifts were organized with three mornings and two afternoons each week, resulting in an average of 14 consultations per shift or approximately seven consultations per pharmacist. Public attention to the call center was promoted on various social media platforms, via local/community WhatsApp groups, and through a radio campaign and printed media.

The consultations at the call center were categorized into three groups: clinical inquiries, logistical inquiries (assistance with locating a medication or a pharmacy, providing information regarding the Israeli medication health basket) and fulfillment of medical rights inquiries. For clinical inquiries, a Singaporean drug-related problem (DRP) classification system [12] and the American Pharmacy Quality Alliance (PQA) Medication Therapy Problem Categories Framework [13] were utilized for further subcategorization based on the nature of the clinical question.

The consultation process commenced with the caller’s identification, requiring full name, ID number, age, sex, and caller HMO (health maintenance organization) information. Subsequently, callers were asked to specify the reason for seeking advice and how they learned about the service. Pharmacists, depending on the complexity and nature of the questions, requested information about the caller’s medication list and medical conditions. To provide appropriate responses, the pharmacists evaluated the caller’s medication profile, referring to drug referential databases and collaborating with other team members as needed.

Patients necessitating follow-up were advised to contact the call center again, while references and recommendations from the consultations were documented.

To identify the primary drugs questioned, we analyzed the main drugs discussed in each consultations using the World Health Organization’s (WHO) Anatomical Therapeutic Chemical (ATC) classification system. This analysis included only those database inquiries with complete medication information.

### Call center business model

The telepharmacy call center is supported by the PSI, a non-profit organization. The PSI oversees the financial aspects of the call center, including pharmacist salaries, documentation management, and the provision of medical information resources. To increase public awareness of this initiative, the PSI has secured funding from the Israeli Ministry of Health.

### Satisfaction survey

To assess the service’s efficacy and perception, an anonymous survey was distributed among a representative sample of callers using the Microsoft 365 Forms platform. The survey was sent to via an SMS message a few days after the patient call to the telepharmacy center.

The survey was sent to a sample of patients after major media publication waves (press and radio) and after there was a larger peak of calls to the telepharmacy call center. it was sent twice during the call center operation – in January and May 2023 – and was sent to patients who contacted the telepharmacy call center in the previous month and a half. The survey was sent only to patients who agreed to provide their mobile phone number during their talk with the pharmacists and was anonymous.

The survey aimed to collect feedback on various aspects, including the purpose of contacting the call center, the perceived empathy and attentiveness of the pharmacists, and the effectiveness of their responses. Additionally, the survey evaluated whether callers would recommend the call center to friends or family, any modifications made to the medication regimen following the consultation, the treating physician’s agreement with the pharmacist’s recommendations (if the patient consulted the physician after the call), the benefits derived from the consultation, and overall satisfaction with the service, rated on a scale of 1 to 10, with 10 representing the highest satisfaction level.

The complete survey questionnaire is available in the article appendix 1.

### Statistical analysis

Descriptive statistics of the data collected in the database were performed using measures of central tendency and dispersion including the mean, median and interquartile range. Continuous variables are expressed as the mean value along with one standard deviation (SD). Categorical variables are presented as percentages.

## Results

Throughout the duration of the operation, the telepharmcy center received a total of 1542 inquiries, 93.3% of which were received via telephone (*n* = 1439). Conversely, a minority of 103 inquiries were received through other channels. Specifically, 68 inquiries (4.4% of total inquiries) were submitted via email exclusively, 10 inquiries (0.64%) involved a combination of telephone and email communication. the method of contact was not documented for 25 inquiries (1.6%).

Table 1 summarizes all the information collected on the calls made to the telepharmacy consultation center.

**Table 1:** Demographic and clinical data of patients utilizing the service

Characteristics	Total inquires (*n* = 1542)
**Demographic characteristics**	
Age in years	
mean, SD	59.08 ± 22.2
Median (IQR)	67 (42–76)
Males (N, %)	493, 31.9%
HMO (N, %)	
Clalit	574, 37.2%
Leumit	56, 3.6%
Mehudet	127, 8.2%
Maccabi	354, 22.9%
Military	4, 0.25%
Unkonwn	427, 27.6%
The channel through which they learned about the service (N, %)	
Radio commercial	207, 13.4%
By word of mouth	187, 12.1%
Newspaper	166, 10.7%
Recommendation for medical personal	128, 8.3%
Facebook	99, 6.4%
Internet search	88, 5.7%
Whatsapp smartphone application	48, 3.1%
Community publication	45, 2.9%
Lecture	11, 0.7%
Unknown	563, 36.5%
**Inquiry characteristics**	
Inquiry type (N, %)	
Clinical	1373, 89.0%
Logistic	115, 7.4%
Patient rights	16, 1.0%
Multiple types	5, 0.3%
Other	14, 0.9%
Response channel (N, %)	
Telephone call	1318, 85.4%
E-mail	64, 4.1%
Whatsapp smartphone application	4, 0.25%
SMS message	2, 0.12%
Multiple methods	51, 3.3%
Unknown	103, 6.6%
Consultation time in minutes (mean, SD)	12.7 ± 8.3

SD = standard deviation; IQR = interquartile range

**Fig. 1:**
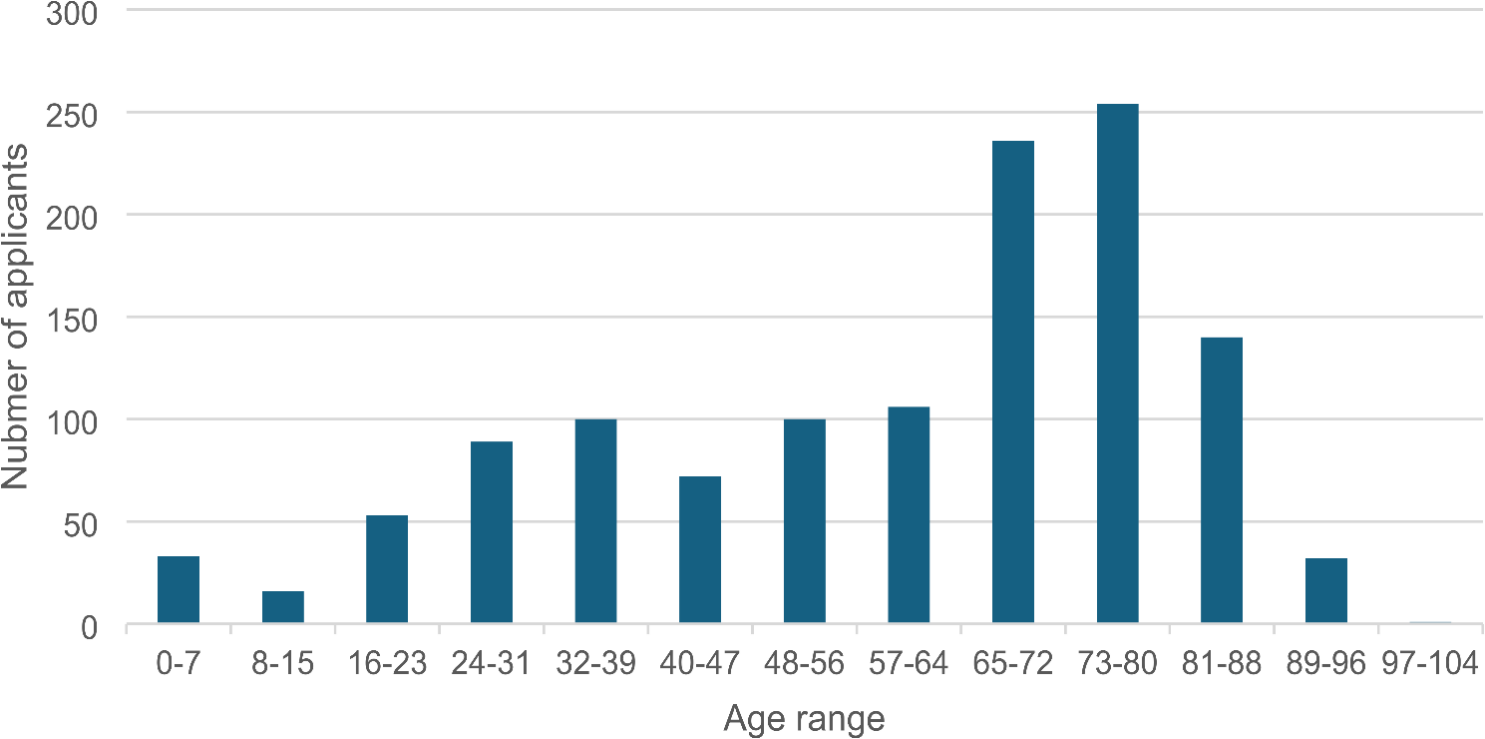
Age range of patients for whom inquiries were made to the telepharmcy call center Detailed legend: None

**Fig. 2:**
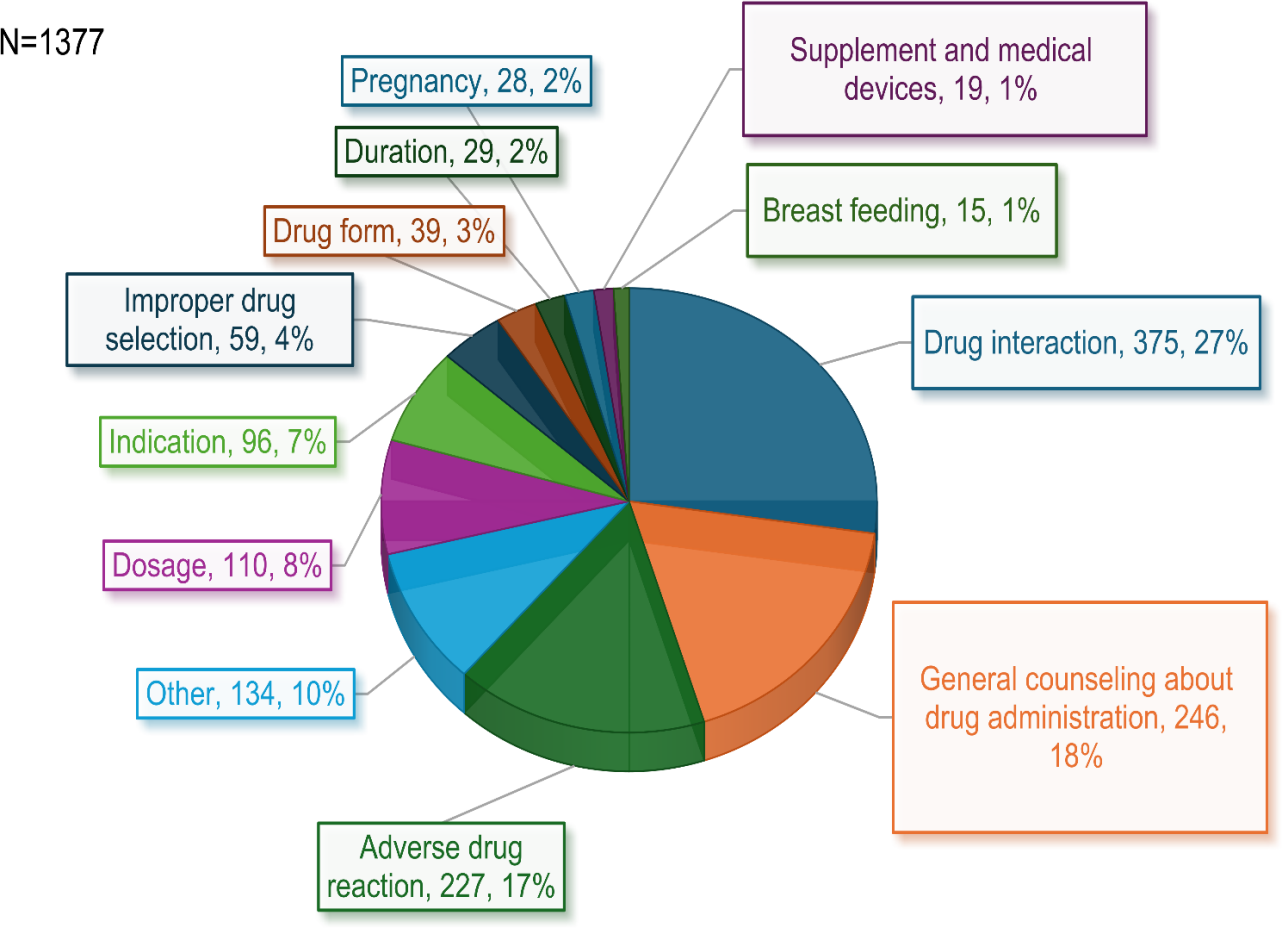
Frequency of clinical query types Detailed legend: None

The age range of callers, or patients whose behalf inquiries were made, spanned from 10 days to 102 years (Fig. 1). Most applicants, accounting for 38.4% (*n* = 593), fell within the 65–85 age range. The predominant category of issues prompting contact with the telepharmcy center was clinical queries, encompassing 89% of inquiries (*n* = 1373), logistical matters comprised only 7.4% (*n* = 115), and patient rights-related concerns represented 1%. Regarding clinical inquiries, 26.7% (*n* = 375) pertained to drug interactions, 17.8% (*n* = 246) addressed general guidance on drug usage, and another 16.4% (*n* = 227) were associated with inquiries about drug side effects (Fig. 2).

An analysis of a random sample of 367 inquiries (23.8% of total inquiries including 614 medications) to the telepharmacy call center revealed that the primary drugs of concern or related to the queries encompassed herbs, vitamins, minerals and nutritional supplements (11.4%), antihypertensive drugs (10.7%) and non-steroidal anti-inflammatory drugs (7%). The complete list is on Table 2. According to the ATC classification, the most common drug classes were ‘nervous system drugs’ (ATC group N) and ‘cardiovascular systemic use’ (ATC group C).

**Table 2:** Distribution of medications involved in pharmaceutical consultations (data from a sample of 367 consultations)

Clinical Query Type	ATC	Number of medications	%
Herbs, vitamins, minerals and nutritional supplements	unclassified	70	11.4
Hypertension medications	C	66	10.7
Non-Steroidal Anti-Inflammatory Drugs (NSAIDS)	M	43	7
Antidepressants	N	38	6.2
Cholesterol-lowering drugs	C	30	4.9
Antibiotics	J	26	4.2
Diabetes mellitus Non-insulin medications	A	25	4.1
Antiplatelet agents	B	24	3.9
Benzodiazepines and Z-drugs	N	21	3.4
Opioids	N	20	3.3
Medications for reducing stomach acid levels	A	18	2.9
Anticoagulants	B	16	2.6
Urologics	G	16	2.6
Antiepileptics	N	13	2.1
Medical cannabis	N	12	2
Antipsychotic drugs	N	12	2
Thyroid hormone replacement	H	12	2
antiarrhythmics	C	11	1.8
ADHD medications	N	11	1.8
Diuretics	C	10	1.6
Mild pain relievers	N	10	1.6
Others	unclassified	110	17.9
**Total**		**614**	**100%**

**Fig. 3:**
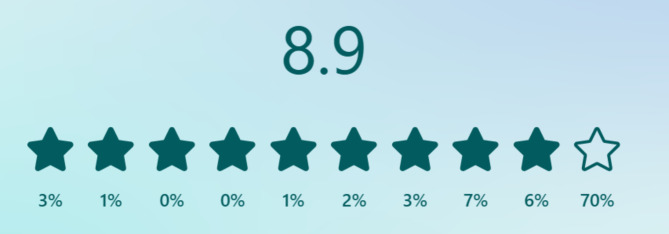
The overall average rating and score distribution for the service (*n* = 158) Detailed legend: From left to right: 1 star - lowest rating and 10 stars - highest rating

The patient satisfaction survey elicited responses from 170 patients (356 patients received the survey via SMS, 47.7% response rate). Primary motivations for choosing the call center included the convenience of calling from home (30%), the availability of the call center and the possibility of receiving unrushed answers and advice (23% for both). A total of 148 respondents (87%) strongly agreed that the pharmacist demonstrated empathy and attentiveness toward their questions. Additionally, 159 respondents (93.5%) either completely agreed or agreed that the pharmacist provided a satisfactory response to their inquiries. Among the respondents, 160 patients (94.1%) expressed their willingness to recommend the center to friends or family members. The service received an average overall rating of 8.9 (Fig. 3). The comprehensive survey results are attached in Additional file 1, Appendix 1.

## Discussion

In this retrospective-descriptive study, we delineated the operations of a large scale telepharmacy center, emphasizing its significance to patients, the varied range of inquiries it received, and the elevated satisfaction levels among those seeking its services. To our knowledge, this marks the first endeavor in Israel to establish a free and accessible telepharmacy center utilized by all HMO patients. Moreover, this is the first study detailing the activities of this center and gauging satisfaction levels in the Israeli context.

Israeli HMOs have previously launched initiatives to create and promote health related call centers. However, none of these initiatives have advanced in the field of pharmacy by staffing the call centers exclusively with pharmacist and dedicated to offering immediate pharmaceutical consultations similar to what a patient could receive at the physical pharmacy.

This study could provide policymakers with valuable information about the feasibility of operating telepharmacy, the value it provides to callers, and the satisfaction of patients who use it.

The call center has proven its value as the primary point of contact for drug-related inquiries, addressing clinical, logistical, and administrative questions, as outlined above. Moreover, patients found it convenient to ask more personalized and comprehensive questions about treatment integration into their daily lives in the stress-free and private settings of their homes.

There are several key points that can be learned from the current research on the benefits of pharmaceutical counseling provided through a nationally spread telepharmacy center.

The duration of counseling is pivotal in the telepharmacy call center setting. In contrast to the average 3.98 min per interaction at the community pharmacy counter [14], the mean call duration was 12.7 min, enabling more comprehensive counseling that covers medical history inquiries, over-the-counter medications, supplements, and detailed explanations of drug administration—far surpassing the brief responses typical in the pharmacy counter setting.

Furthermore, the prevalence of patients seeking pharmacist support for drug interactions, counseling, and adverse reactions underlines the pharmacists valuable role in optimizing medication use and mitigating risks. This highlights the need for further empowering pharmacists within healthcare delivery systems.

Moreover, unlike the lack of confidentiality observed in staff-patient conversations within a pharmacy setting [15], the call center facilitated private counseling, allowing patients to share medical information at their discretion. We propose that this call center model could be replicated for patients requiring a higher level of confidentiality, such as individuals living with HIV, or for populations with elevated religious or social constraints, such as the ultraorthodox Jewish or Muslim communities in Israel [16].

The preponderance of inquiries originated from or concerned patients over 60 years old may highlight the demand among elderly people for detailed and extended pharmaceutical counseling and guidance. This needs to be proven in a dedicated study. We posit that similar initiatives will be most advantageous for this age group, given the age-related surge in chronic diseases and medication use.

A cross sectional study by Reed et al. questioned Which patient characteristics are associated with choosing either a telemedicine visit or an office visit with the same primary care clinician [17]. They concluded that Telemedicine may offer the potential to reach vulnerable patient groups and improve access for patients with transportation, parking, or cost barriers to clinic visits. These challenges indeed characterize the elderly population and are therefore likely to be relevant in the context of telepharmacy as well.

Patient satisfaction with the call center was high and consistent with the findings of other studies that have examined this issue.

Moulaei et al. explored patients’ perspectives on telepharmacy as an alternative to traditional in-person visits, and revealed that 77% of respondents preferred telepharmacy, particularly for reasons such as reducing the incidence of contagious diseases, saving time, and cutting costs, indicating its potential importance during crises such as the COVID-19 pandemic [18].

Ho et al. described a nationwide online telepharmacy chat service in Denmark. This study analyzed 500 consecutive chat transcripts, and revealed diverse enquiries categorized into drug-related (35.7%), technical (26.1%), symptom-related (19.1%), and other (19.1%) categories. Of the customers, 89.2% were satisfied with the online counseling [19].

Guénette et al. evaluated the “Ask Your Pharmacist” teleconsultation platform in Quebec, Canada, through a satisfaction survey of 53 patients and 27 pharmacists, revealing high satisfaction levels among patients (96.2%) and positive motivations for pharmacists, such as meeting patient needs (85.1%). Patients indicated that the platform might reduce the need for additional consultations and visits to physicians or emergency departments, emphasizing the potential usefulness of teleconsultation services provided by pharmacists in enhancing healthcare accessibility [20].

An additional noteworthy finding was the greater influence of traditional media compared to social media in promoting awareness of the PSI telepharmacy call center. This disparity may be attributable to differences in patient demographics (e.g., age, socioeconomic status). The telepharmacy call center experienced a surge in call volume following promotional campaigns on radio and in print media.

Moreover, telepharmacy call center utilization rates across HMOs were generally proportional to their respective patient populations in Israel. HMOs planning to implement or expand telepharmacy services should consider these observations when targeting specific patient groups and estimating service demand.

In our study, we observed that clinical inquiries were the most common type of questions posed by the Israeli public. The predominant category of queries concerned dietary supplements and medicinal herbs, reflecting a significant gap in information for both healthcare professionals and patients in this area. Regarding conventional medications, the most frequent inquiries focused on drugs for treating cardiovascular diseases, particularly hypertension and hypercholesterolemia, as well as commonly used medications for inflammatory conditions, depression, and antibiotics. These trends align with the primary drug-related questions addressed in a Danish tele-pharmacy center study [19]. However, unlike our findings, the Danish center reported a greater volume of inquiries about drugs within the ATC category of the genitourinary system and sex hormones.

The experience of operating a telepharmacy center has also revealed several challenges that need to be considered.

The absence of access to patient medical records and medical history poses a challenge for the call center, but in most cases, a detailed response was feasible. Patient adherence was self-reported and not corroborated by medical records. This reliance on self-reported data, with its potential for missing or inaccurate information, could lead to inaccurate counseling and potential medicolegal risks.

We believe that access to medical records could enhance the efficacy of calls, providing more precise responses tailored to individual patients.

The absence of a centralized pharmaceutical inventory database in Israel limited the information pharmacists at the call center could provide. While Meuhedet, Clalit, and Maccabi HMOs, along with Super-Pharm (a major drugstore chain), offer some external access to their inventories, this access is either limited to specific pharmacies (Super-Pharm) or unavailable (Leumit HMO, other drug store chains, and independent pharmacies). Consequently, patients may need to contact multiple pharmacies to determine medication availability.

The inability to directly communicate potential drug-related issues to prescribing physicians limited our ability to assess the impact of patient counseling. For future telepharmacy call centers, establishing direct communication with physicians is crucial. This would not only allow for real-time consultation prior to prescribing but also potentially prevent adverse events and inappropriate medication use by providing physicians with immediate access to pharmacist expertise.

A potential gap exists in pharmacists’ training regarding the provision of emotional and mental health support, particularly for patients in severe distress. Pharmacists in telepharmacy call centers could benefit from additional training in this area.

A significant obstacle has been the lack of funding, despite the potential clinical and social benefits. While the Ministry of Health (MOA) supported the call center’s promotion through mass media for a limited time, the operation itself was funded by PSI. Recognizing the importance of holistic pharmaceutical counseling, the private practices of pharmacists in Israel now offer thorough comprehensive medication management (CMM) using patient-provided medical history and records. The implementation of CMM has been associated with higher rates of achievement of treatment goals for a wide range of chronic diseases [21]. Similarly, counseling services, whether through a telephone call center or face-to-face interactions, should be accessible to the general public under the NHB list to potentially minimize socioeconomic gaps, reduce mortality, and prevent further drug-related problems (DRPs).

Furthermore, the impact of telepharmacy on patient safety, clinical outcomes, as well as its effects on pharmacist workload, morale, and attrition, as emphasized by Unni et al. [5], requires thorough evaluation.

For example, a systematic review on community pharmacy-based telepharmacy services reported uncertain results about their safety and quality. Among the 866 studies, only six met the inclusion criteria, and the outcomes varied. There was no clear difference in medication safety and adherence, conflicting evidence on patient satisfaction, and insufficient data on inappropriate medication use. A high risk of bias prevented definitive conclusions, emphasizing the need for stronger study designs and rigorous methodologies to establish conclusive evidence on the effectiveness of these services [22]. Le et al. in a literature review of telepharmacy in community and ambulatory pharmacy settings in the United States suggested that telepharmacy is likely to continue expanding because it offers better resource allocation and increased patient access, but emphasizes the need for further research to analyze the specific value and role of telepharmacy services [23].

Another point is the acceptance of telepharmacy by the pharmacists themselves, which is significantly different from their current face-to-face work model. A cross-sectional survey of 136 Canadian pharmacists found that the utilization of telepharmacy is relatively low, despite the growing recognition of its benefits. Among pharmacists using telepharmacy, the majority felt that telepharmacy enhanced their clinical practice, while nonusers believed it would benefit their practice. This study suggests areas of consideration for better integration of telepharmacy in pharmacy practice, including optimizing workflows, addressing barriers, and providing training to pharmacy students [24].

The role of pharmacists within Israel’s healthcare system has evolved considerably in recent decades. Historically focused on extemporaneous compounding, the advent of mass-produced medications has altered pharmacists’ daily responsibilities, contributing to a public perception of pharmacists as primarily dispensers, despite their substantial clinical and academic expertise. Community pharmacists participating in the PSI telepharmacy call center report that their routine duties at pharmacy counters are often perceived by patients as largely logistical or driven by commercial considerations, particularly concerning over-the-counter medications. Importantly, all participating pharmacists cited the opportunity to provide clinical advice as their primary motivation for call center involvement.

Two key challenges contribute to this perception. First, a lack of awareness exists among policymakers, healthcare providers, and the public regarding pharmacists’ expanded roles. Second, the heavy workload associated with dispensing, limits time available for other clinical activities, thereby restricting public exposure to other pharmacists capabilities [25].

Despite the potential for pharmacists to be viewed as non-clinicians within the call center context, this study demonstrates a significant paradigm shift. The majority of inquiries received (89%) were of clinical significance, rather than logistical or related to patient rights concerning medication access. This finding supports the authors’ hypothesis that the public image of pharmacists in Israel can evolve from that of mere dispensers to integral members of the healthcare team, by providing opportunities for pharmacists to deliver clinical expertise directly to patients.

Given the critical role pharmacists play in medication management, patient counseling, and education, this work strongly supports the inclusion of CMM as a reimbursable service within the NHB. Pharmacists are uniquely positioned to enhance medication literacy, improve adherence, and ultimately contribute to better patient outcomes. Including pharmacist consultation services in the NHB could address pressing issues within the Israeli healthcare system, such as improving medication literacy and reducing drug-related problems [26, 27].

In 2022, the PSI applied to include pharmacist counseling services, based on CMM principles, in the NHB. The application was rejected, based on the premise that such counseling is already included in the NHB as standard practice within community pharmacies. This is despite the key differences between CMM and counter pharmaceutical counseling, particularly in resource requirements. Furthermore, unlike other NHB-covered services (e.g., physiotherapy, emergency care), HMOs have not actively promoted this service to patients.

Following the initial rejection, the PSI and other pharmacists’ organizations petitioned the High Court of Justice, requesting that HMOs be directed to actively promote the service to eligible patients and establish clear eligibility criteria.

Future initiatives should focus on advancing telepharmacy as a significant service in Israel. This entails developing a robust communication platform, enhancing education and qualifications for pharmacists, and increasing patient awareness of the service. Additionally, investigating the social impact, including the public perception of telepharmacy and technology access, while ensuring the security and integrity of patient data during the electronic transmission of health information, is of utmost importance.

### Study limitations

Our study has several limitations. The consultation solely addressed the applicant’s immediate query, precluding assessment of broader clinical outcomes or long-term implementation of recommendations. The pharmacists did not possess access to patient medical records, relying solely on the applicant’s self-reported information during the call. This restricted their ability to provide holistic, contextualized interventions, lacking insights into existing medications and potential drug interactions.

Additionally, no documented follow-up was performed to assess the completion of the referral (e.g., confirmation with the attending physician). The short operational period (eight months, primarily fall and winter) of the telepharmacy center introduces a potential seasonal skew in inquiry types (e.g., increased winter inquiries about over-the-counter remedies). This limits the generalizability of the findings across seasons.

Medication data from each inquiry was documented beginning 3 months after the call center launch, and some records incompletely documented all medications discussed. As a result, a detailed breakdown of medications related to consultations was available for only 367 inquiries. Nevertheless, we believe this represents a sufficiently representative sample (nearly 24% of total inquiries), making it unlikely that the percentage breakdown of medications involved would significantly differ had data from all inquiries been included. Finally, the anonymous nature of the survey precluded the performance of a regression analysis, thereby limiting the identification of factors associated with patient satisfaction.

These limitations highlight the need for further research investigating the long-term impact of pharmacist-led telephone counseling (telepharmacy) on both clinical outcomes and patient adherence to recommendations. Future studies should consider models that grant pharmacists-controlled access to patient records to enhance the quality and scope of their consultations. Addressing these limitations can refine and expand the potential benefits of pharmacist-led telephone consultations within the healthcare landscape.

The PSI telepharmacy call center, initially closed in June 2023 due to the completion of a pilot program and funding, was reopened on October 9, 2023, just two days after the outbreak of the ‘Iron Swords’ war in Israel. During this period, the call center operated with extended hours and a larger team of 107 volunteer pharmacists. It remained open until November 31, 2023, receiving a total of 1148 inquiries. Once HMOs established medicine delivery services and call volume decreased, the call center was closed again. Since March 17, 2024, the call center has been operating in its original format with 8 pharmacists, supporting both healthcare providers and the public.

## Conclusion

Delivering pharmacy services through telepharmacy in Israel has proven to be both feasible and well-received, garnering high satisfaction levels among applicants. This innovative service enhances accessibility and is anticipated to minimize gaps in community access to pharmaceutical counseling, particularly for older adults.

This paper highlights the critical need for an accessible and well-promoted telepharmacy center.

## Electronic supplementary material

Below is the link to the electronic supplementary material.

Additional file 1. Appendix 1. The comprehensive survey results.

## Data Availability

The datasets analyzed during the current study available from the corresponding author on reasonable request.

## List of abbreviations

ASHP: The American Society of Health System Pharmacists
ATC: Anatomical Therapeutic Chemical
CMM: Comprehensive Medication Management
DRP: Drug-related problem
HMO: Health Maintenance Organization
MOH: Ministry of health.
PSI: Pharmaceutical Society of Israel
PQA: Pharmacy Quality Alliance
