# Correction: Enhancing medication literacy through a telepharmacy call center in Israel: consultation overview and patient satisfaction

**DOI:** 10.1186/s13584-026-00760-5

**Published:** 2026-05-22

**Authors:** Ran Nissan, Rana Cohen, Maria Hurgin, Hen Popilski, Khaleel Zahalka, Merav Ben Natan, Eyal Schwartzberg

**Affiliations:** 1 Pharmaceutical Society of Israel, Tel Aviv, Israel; 2 Division of Clinical Pharmacy, Institute for Drug Research, School of Pharmacy, The Hebrew University, Jerusalem, Israel; 3 Pat Matthews Academic School of Nursing, Hillel Yaffe Medical Center, Eyal Schwartzberg, Hadera, 26736 Israel; 4 School of Pharmacy, Community Clinical Pharmacy and Regulatory Management MSc Program, Ben Gurion University, Beer Sheva, Israel

**Correction: Israel Journal of Health Policy Research (2025) 14:26**

10.1186/s13584-025-00686-4

The original publication of this article contained an incorrect author name. The incorrect and correct information is listed in this correction article, the original article has been updated.

Incorrect

Meirav Ben Natan.

Correct

Merav Ben Natan.

