# Supplementary material for: Enhancing medication literacy through a telepharmacy call center in Israel: consultation overview and patient satisfaction
Source: Isr J Health Policy Res. 2025 May 1;14:26. doi: 10.1186/s13584-025-00686-4 (PMC12046693; doi:10.1186/s13584-025-00686-4)
Supplement: Supplementary file 1 — Additional file 1. Appendix 1. The comprehensive survey results. [file 13584_2025_686_MOESM1_ESM.docx]

**Appendix 1 - Enhancing Medication Literacy: A retrospective analysis of the telepharmacy call center impact on patient care in Israel**

Comprehensive patient satisfaction survey results

**
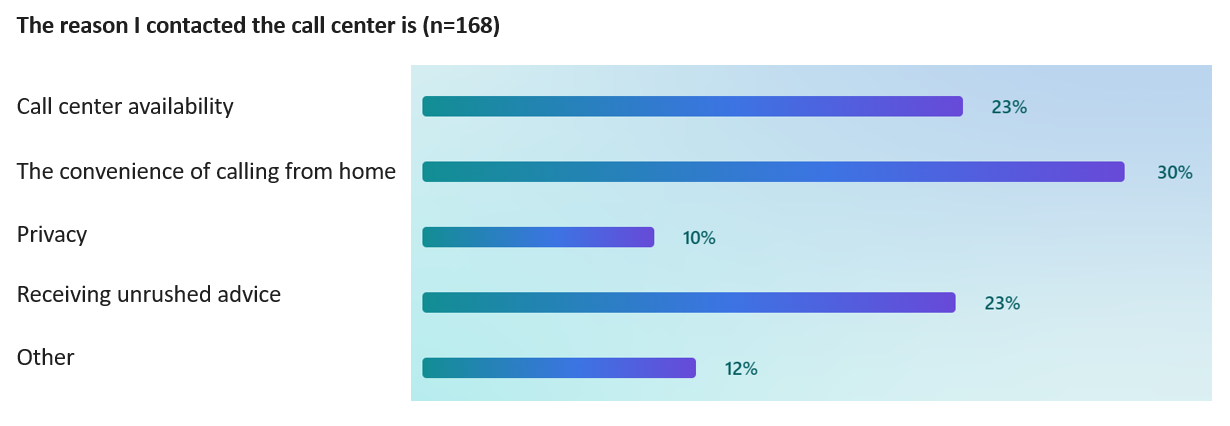
**

**
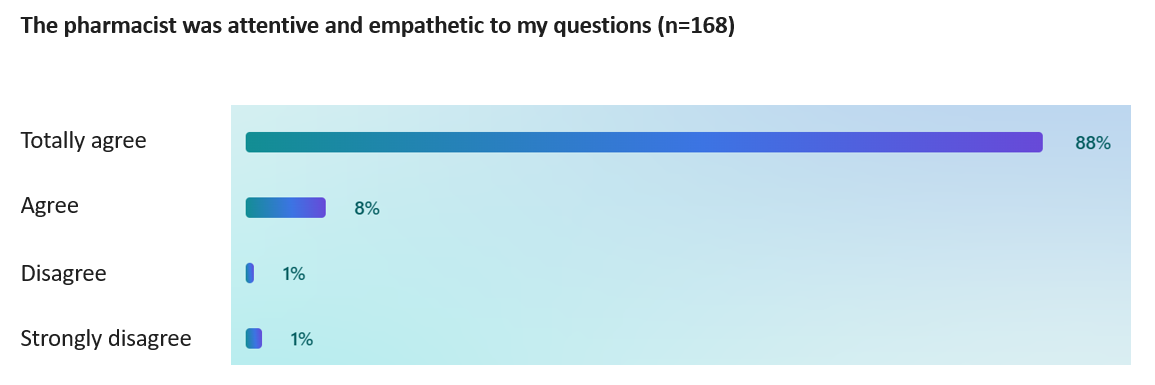
**

**
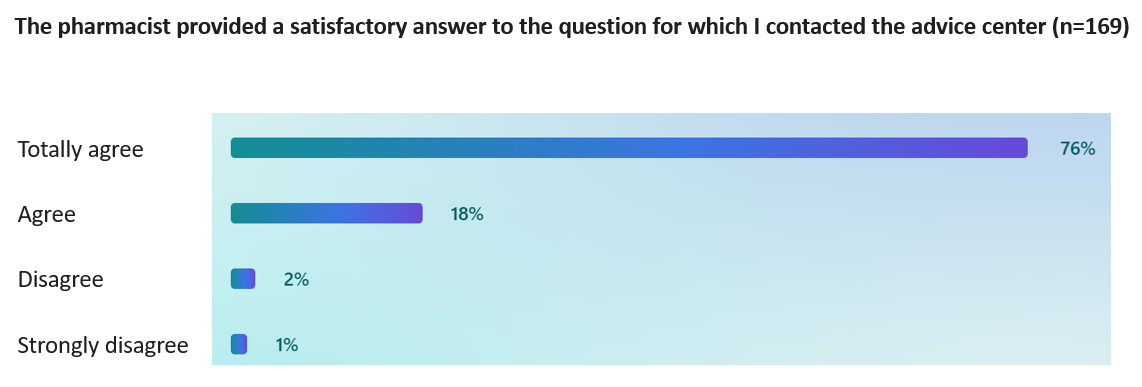
**

**
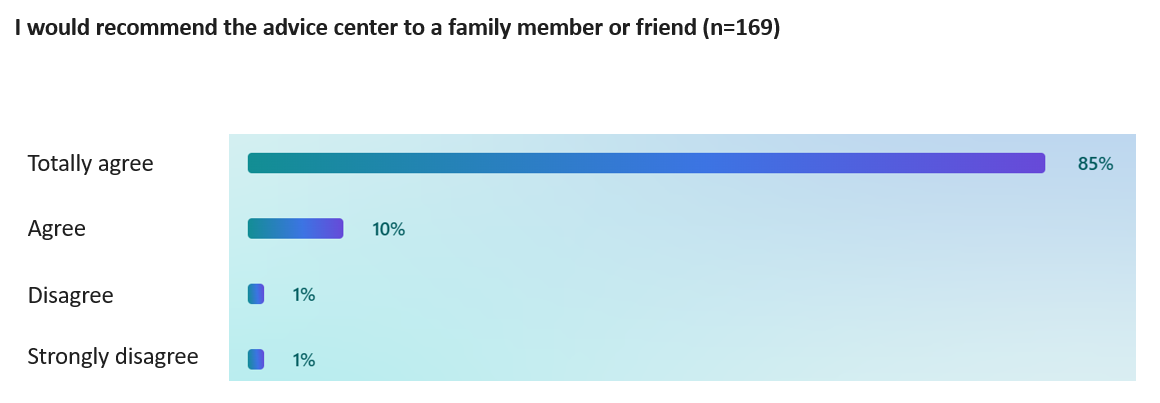
**

**
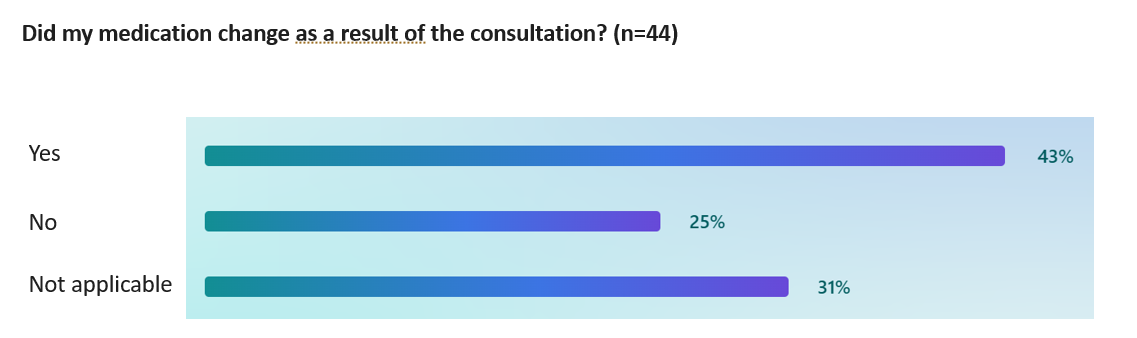
**

**
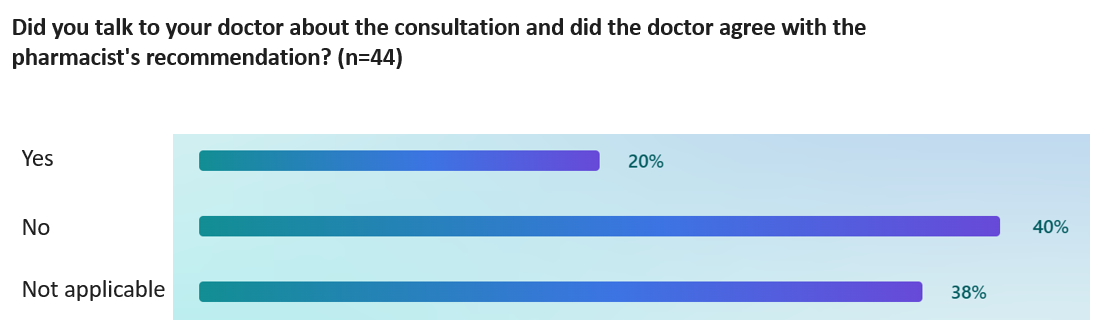
**

**
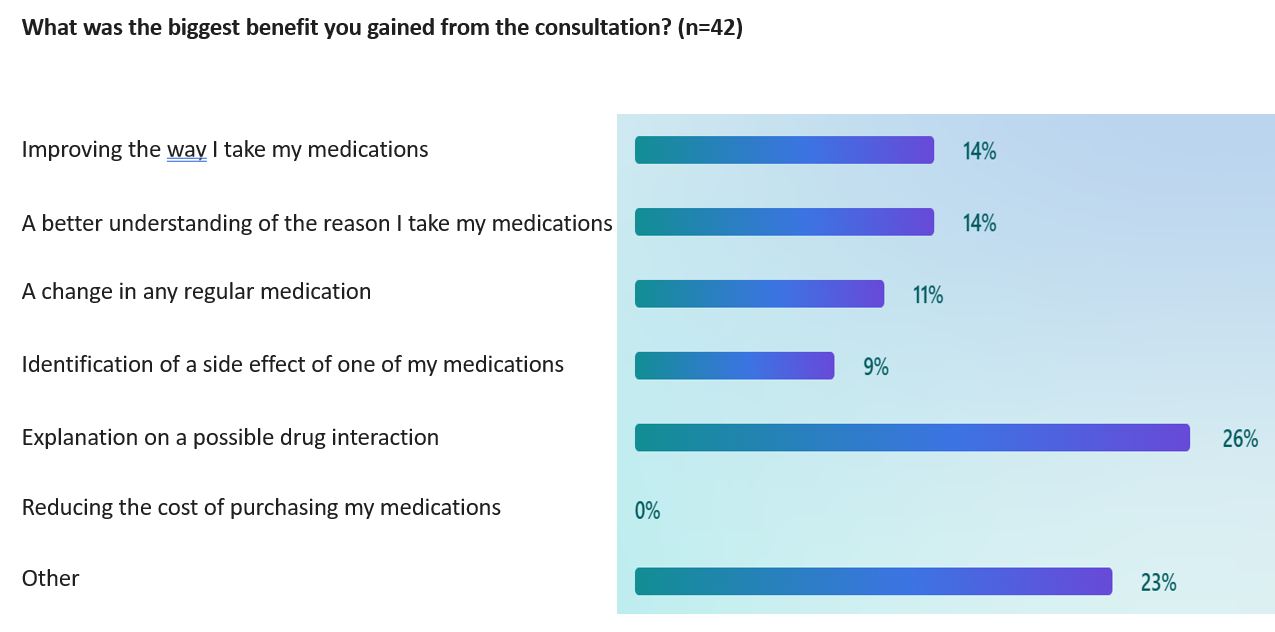
**
